# Optimized polyepitope neoantigen DNA vaccines elicit neoantigen-specific immune responses in preclinical models and in clinical translation

**DOI:** 10.1186/s13073-021-00872-4

**Published:** 2021-04-21

**Authors:** Lijin Li, Xiuli Zhang, Xiaoli Wang, Samuel W. Kim, John M. Herndon, Michelle K. Becker-Hapak, Beatriz M. Carreno, Nancy B. Myers, Mark A. Sturmoski, Michael D. McLellan, Christopher A. Miller, Tanner M. Johanns, Benjamin R. Tan, Gavin P. Dunn, Timothy P. Fleming, Ted H. Hansen, S. Peter Goedegebuure, William E. Gillanders

**Affiliations:** 1 Department of Surgery, Washington University School of Medicine, 660 South Euclid Avenue, St Louis, MO 63110 USA; 2 Department of Pathology and Immunology, Washington University School of Medicine, St Louis, MO USA; 3 Department of Medicine, Washington University School of Medicine, St Louis, MO USA; 4 Present Address: Parker Institute for Cancer Immunotherapy, Center for Cellular Immunotherapies, Perelman School of Medicine, University of Pennsylvania, Philadelphia, PA USA; 5 McDonnell Genome Institute, Washington University School of Medicine, St Louis, MO USA; 6 The Alvin J. Siteman Cancer Center at Barnes-Jewish Hospital and Washington University School of Medicine, St Louis, MO USA; 7 Present Address: Norton Thoracic Institute, St. Joseph Hospital and Medical Center, Phoenix, AZ USA

**Keywords:** Neoantigen, Polyepitope DNA vaccine, Breast cancer

## Abstract

**Background:**

Preclinical studies and early clinical trials have shown that targeting cancer neoantigens is a promising approach towards the development of personalized cancer immunotherapies. DNA vaccines can be rapidly and efficiently manufactured and can integrate multiple neoantigens simultaneously. We therefore sought to optimize the design of polyepitope DNA vaccines and test optimized polyepitope neoantigen DNA vaccines in preclinical models and in clinical translation.

**Methods:**

We developed and optimized a DNA vaccine platform to target multiple neoantigens. The polyepitope DNA vaccine platform was first optimized using model antigens in vitro and in vivo. We then identified neoantigens in preclinical breast cancer models through genome sequencing and in silico neoantigen prediction pipelines. Optimized polyepitope neoantigen DNA vaccines specific for the murine breast tumor E0771 and 4T1 were designed and their immunogenicity was tested in vivo. We also tested an optimized polyepitope neoantigen DNA vaccine in a patient with metastatic pancreatic neuroendocrine tumor.

**Results:**

Our data support an optimized polyepitope neoantigen DNA vaccine design encoding long (≥20-mer) epitopes with a mutant form of ubiquitin (Ub^mut^) fused to the N-terminus for antigen processing and presentation. Optimized polyepitope neoantigen DNA vaccines were immunogenic and generated robust neoantigen-specific immune responses in mice. The magnitude of immune responses generated by optimized polyepitope neoantigen DNA vaccines was similar to that of synthetic long peptide vaccines specific for the same neoantigens. When combined with immune checkpoint blockade therapy, optimized polyepitope neoantigen DNA vaccines were capable of inducing antitumor immunity in preclinical models. Immune monitoring data suggest that optimized polyepitope neoantigen DNA vaccines are capable of inducing neoantigen-specific T cell responses in a patient with metastatic pancreatic neuroendocrine tumor.

**Conclusions:**

We have developed and optimized a novel polyepitope neoantigen DNA vaccine platform that can target multiple neoantigens and induce antitumor immune responses in preclinical models and neoantigen-specific responses in clinical translation.

**Supplementary Information:**

The online version contains supplementary material available at 10.1186/s13073-021-00872-4.

## Background

Cancer neoantigens are created by somatic DNA alterations resulting in protein sequence changes capable of triggering adaptive immune responses. Next generation sequencing, together with bioinformatics-based computational algorithms, has revolutionized our ability to identify cancer neoantigens [1, 2]. We and others have demonstrated that cancer neoantigens are important targets during cancer immunoediting and that cancer sequencing combined with epitope prediction algorithms can be used to identify and prioritize neoantigens for integration into personalized cancer vaccines [3–5]. Conceptual advantages associated with cancer vaccines targeting cancer neoantigens include the fact that neoantigens are not found in normal tissues, decreasing the risk of autoimmunity and/or central immune tolerance.

Neoantigen vaccines based on the synthetic long peptide (SLP), RNA, and dendritic cell (DC) platforms appear to be capable of inducing neoantigen-specific T cell responses, and potential favorable clinical outcomes [6–11]. In order to maximize antitumor immunity and to prevent or curtail tumor immune escape, targeting multiple neoantigens simultaneously is desirable. However, manufacturing neoantigen vaccines based on the SLP, RNA, or DC vaccine platforms under good manufacturing practice (GMP) conditions is both time consuming and resource-intensive. In comparison, one of the strengths of the recombinant DNA vaccine platform is the relative ease of manufacture of plasmid DNA on a scale appropriate for personalized vaccines. As such, the recombinant DNA vaccine platform represents an attractive platform for the clinical development of polyepitope neoantigen cancer vaccines.

Advantages of the DNA vaccine platform include its remarkable safety profile, the relative ease of manufacture, and the molecular flexibility that allows integration of multiple neoantigens using a single polyepitope construct. Recent advances in the DNA vaccine platform, such as gene/vector optimization, molecular/formulation adjuvants, and DNA delivery by electroporation, have significantly improved the efficacy of DNA vaccines, and numerous early phase clinical trials are ongoing in the infectious disease and cancer fields [12]. We report here our efforts to optimize the polyepitope neoantigen DNA vaccine in preclinical models to maximize neoantigen presentation and vaccine immunogenicity. We addressed the following questions: (1) are longer epitopes (≥20-mers) processed equally well as minimal epitopes (e.g., 9-mers); (2) will short flanking sequences (spacers) between epitopes enhance antigen processing and reduce creation of junctional epitopes; and (3) will the addition of a mutant form of ubiquitin enhance neoantigen processing and presentation? Our study demonstrates that polyepitope inserts encoding 20–25-mer neoantigen epitopes (with or without spacers) fused with a mutant form of ubiquitin are efficiently processed and presented. Model DNA vaccines designed with this strategy were able to induce immune responses in vivo, and neoantigen DNA vaccines were able to induce antitumor immune responses in preclinical breast cancer models and neoantigen-specific T cell responses in clinical translation.

## Methods

### Animals

Female C57BL/6J (H-2^b^) and Balb/cJ (H-2^d^) mice were purchased from the Jackson Laboratory (Bar Harbor, ME). HHD II transgenic mice [13] were originally obtained from Dr. F. Lemonnier (Institut Pasteur, Paris, France) and were maintained in SPF animal facilities. These mice express the transgene Tg (HLA-A/H2-D/B2M) 1Bpe in a mixed background involving B2M^tm1Unc/tm1Unc^ and H2-D1^tm1Bpe/tm1Bpe^. They express chimeric MHC-I heavy chain with HLA-A*0201 (α1-α2) and H-2D^b^ (α3-transmembrane and intracytoplasmic domains), allowing the study of HLA-A2-restricted responses in vivo. All animals were used at 7–10 weeks of age. Protocols were approved by the Animal Studies Committee of Washington University School of Medicine (WUSM) and were in accordance with IACUC guidelines and procedures.

### Tumors and cell lines

HeLa cells that stably express HLA-A2 (HeLa-A2), murine and human TAP-deficient RMA-S (H-2^b^) and T2 cells made to express mouse MHC class I molecules were obtained from Dr. T. Hansen (Washington University School of Medicine). E0771 [14] and 4T1.2 [15] are mouse breast cancer cell lines of C57BL/6 (H-2^b^) and Balb/c (H-2^d^) origin, respectively. All cells were cultured in RPMI-1640 complete media (Gibco) supplemented with L-glutamine, 10% fetal bovine serum (Atlanta Biologicals, Flowery Branch, GA), sodium pyruvate, non-essential amino acids, and penicillin-streptomycin (Gibco).

### Genome sequencing and neoantigen identification

Genomic DNA and RNA were extracted from E0771 and 4T1.2 tumors grown in vivo using commercially available kits (Qiagen). Tails from C57BL/6 and Balb/c mice were used as normal control. Exome and cDNA-capture sequencing were performed as previously described [3, 5]. The pVACseq pipeline, version 1.0.0-beta [16, 17], was applied to identify genetic alterations and prioritize neoantigens based on the tumor/normal sequencing data. Briefly, each genetic alteration resulting in an amino acid change was evaluated in the context of 10–14 flanking amino acids. All sub-peptides containing the substitution were evaluated using the suite of IEDB-provided HLA class I peptide-binding algorithms (netMHC, NetMHCcons, netMHCpan, PickPocket, SMM, and SMMPMBEC). In order to prioritize neoantigen candidates for the study, results with median predicted binding affinities (IC_50_) less than 500 nm were filtered based on sample purity (both tumor VAF and RNA VAF > 30%), gene expression level (FPKM > 1), and ranked according to the fold change (MT/WT) of IC_50_ scores. Neoantigens with MT/WT fold change greater than 2 were incorporated in the polyepitope neoantigen DNA vaccines.

### Polyepitope DNA and SLP vaccines

Codon-optimized DNA fragment encoding polyepitope neoantigens were synthesized by Blue Heron Biotech (Bothell, WA) or GenScript (Piscataway, NJ) and subsequently cloned into the mammalian expression plasmid pcDNA 3.1^(+)^ (Invitrogen, Carlsbad, CA) or the pMSV.IRES.GFP (pMIG) retroviral expression vector. The sequences of the polyepitope constructs can be found in Additional file 1. Where indicated, DNA sequence for Ub^mut^, a mutated (G76V) ubiquitin [18], was fused to the N-terminus of the polyepitope construct by standard molecular subcloning. Plasmid DNA were amplified in *Escherichia coli* DH5α (Invitrogen) and purified using NucleoBond Maxi Plasmid DNA Purification kits (Macherey-Nagel, Bethlehem, PA). DNA vaccination was performed using a Helios gene gun (Bio-Rad, Hercules, CA) as previously described [19]. Typically, 4 μg of DNA was delivered to non-overlapping shaved and depilated mice abdominal areas at 3-day intervals (days 0, 3, and 6) for a total of three doses [20]. The discharge helium pressure was set to 400 p.s.i. Immune responses were measured 5 days after the last gene gun vaccination (day 11).

SLPs containing the identified neoantigens were custom-made by GenScript and Peptide 2.0 (Chantilly, VA). Lyophilized peptides were first dissolved in H_2_O or DMSO and stored at − 20 °C. One hundred micrograms of each peptide was diluted in PBS and mixed with 50 μg of poly(I:C) (InvivoGen) before subcutaneous injection on day 0 and day 7. Immune responses were measured by ELISpot assay on day 12.

### Immunoprecipitations and immunoblots

HeLa-A2 cells were transduced with a retroviral vector pMIG [21] encoding polyepitope antigen. GFP^+^ cells were FACS-sorted and cultured for 24 h with or without 50 μM MG132 (Boston Biochem, Cambridge, MA). Expression of GFP protein was also confirmed by western blot of total cell lysate with anti-GFP antibody (Santa Cruz, Dallas, TX). To detect the production and degradation of polyepitopes, immunoprecipitation and immunoblot were performed as previously described [22]. Briefly, cells were lysed in PBS with 1% Nonidet P-40. Post-nuclear lysates were then incubated with anti-HA-Sepharose (Covance). After washes, precipitated proteins were eluted by boiling in LDS sample buffer (Invitrogen) and separated by SDS-PAGE. Proteins were detected with anti-HA (clone 16B12, Santa Cruz) and visualized by chemiluminescence using the ECL system (ThermoFisher).

### Flow cytometry

To measure cell surface expression of neoantigens, a TCR mimic Ab (TCRm) specific for SVG9/HLA-A2 [23] was used to stain HeLa-A2 cells transduced with polyepitope DNA. As a positive control, parental HeLa-A2 cells were incubated with 10 μM SVG9 peptide for the last hours before cell wash. PE-conjugated goat anti-mouse Ig Ab (BD Biosciences, San Jose, CA) was used as secondary Ab. Data from viable cells, gated by forward and side scatter, were acquired on a FACSCalibur (BD Biosciences) and analyzed using FlowJo v10 software (TreeStar, Ashland, OR).

### CTL assay

In vitro CTL assays were performed as previously described [23]. Briefly, target cells (transduced HeLa-A2) were labeled with 0.2 mCi of [^51^Cr] (PerkinElmer, Wellesley, MA) and incubated with SVG9-specific T cells generated from WNV-KUN-immunized HHDII spleen cells. Parental HeLa-A2 cells with or without SVG9 peptide were used as controls. Maximum lysis was achieved by adding 5% Triton-X 100 (Sigma-Aldrich) to the wells. Spontaneous lysis was determined with cultured target cells without CTLs. Supernatants were collected and read by an Isomedic γ-counter (ICN Biomedicals, Huntsville, AL). The specific lysis was calculated by the formula: 100 × [(experimental ^51^Cr release − control ^51^Cr release)/(maximum ^51^Cr release − spontaneous ^51^Cr release)].

### Tumor challenge and TIL analysis

E0771 and 4T1.2 tumor cells were dislodged with Trypsin/EDTA (ThermoFisher) and washed twice with Ca^2+^/Mg^2+^-free PBS. 10^6^ cells were injected subcutaneously into the flanks of female mice. Tumor sizes were measured using an electronic caliper. For checkpoint blockade, 200 μg of anti-PD-L1 (clone 10F.9G2) or isotype control (clone LTF-2) antibody (both from Bio X Cell, West Lebanon, NH) was administered *i.p.* at the indicated time points.

To study the neoantigen-specific T cells present in the tumor after DNA vaccination, tumors were harvested and digested with Tumor Dissociation Kit (Miltenyi Biotec) following the manufacturer’s instruction. Single cell suspensions were prepared by passing through 70-μm cell strainers after cell debris was removed and red blood cells were lysed. Tumor-infiltrating leukocytes were stained with dextramer and analyzed by flow cytometry.

### Tetramer/dextramer staining

PE-conjugated SVG9/HLA-A*0201 tetramer was obtained from the National Institute of Allergy and Infectious Diseases tetramer facility (Emory University, Atlanta, GA). APC-conjugated Lrrc27/H-2D^b^ dextramer was manufactured by Immudex (Copenhagen, Denmark). Cells were stained with tetramer or dextramer for 40 min at 37 °C. Fluorophore-labeled antibodies specific for surface markers (CD45, CD3e, and CD8α) were subsequently added and the cells were incubated for an additional 20 min at 4 °C. Cells were acquired on a FACSCalibur and data were analyzed with FlowJo v10 software.

### Human subject

Patient GTB16 was a 25-year-old male with Lynch Syndrome-associated metastatic pancreatic neuroendocrine tumor that was refractory to standard of care treatment. He was initially diagnosed and treated at Barnes-Jewish Hospital, St. Louis, MO. He received palliative carboplatin/etoposide and concurrent lanreotide with a partial response. Because his tumor demonstrated microsatellite instability, he was also treated with pembrolizumab as maintenance therapy on a compassionate use protocol with a partial response. Repeat surveillance MRI demonstrated mixed response with evidence of ongoing progression. Due to the lack of any effective treatment options available, he was treated with a neoantigen DNA vaccine (pGTB16) on a compassionate use basis. The protocol was approved by the Washington University School of Medicine Institutional Review Board, Institutional Biosafety Committee, and the Food and Drug Administration. Written informed consent was signed by the patient for the treatment and associated research studies. A total of three vaccinations with at least 21 days in between injections were administered. pGTB16 vaccine was delivered intramuscularly using an integrated electroporation device (TDS-IM system, Ichor Medical Systems). Blood was drawn pre- and post-vaccination and peripheral blood mononuclear cells (PBMC) were isolated by Ficoll-Paque PLUS (GE Healthcare) density centrifugation and cryopreserved. PBMCs were used in IFN-γ ELISpot assay to evaluate the generation of a neoantigen-specific immune response.

### ELISpot assay

IFN-γ ELISpot^PLUS^ Kits (Mabtech, Cincinnati, OH) were used as instructed by the manufacturer to measure the in vivo neoantigen-specific immune response. For preclinical studies, mouse spleen or lymph node cells were typically seeded at 2–4 × 10^5^ per well in triplicates. Neoantigen (MT) and wildtype (WT) counterparts were synthesized by Peptide 2.0 or GenScript and were used at the indicated final concentration. For clinical studies, cryopreserved PBMCs were thawed and cultured for 12 days in the presence of human IL-2 (50 U/mL) and 25 μM each of the pooled overlapping peptides (each pool contained two mutated genes). After an overnight rest in culture medium without peptides and IL-2, 10^5^ of the in vitro-stimulated cells were co-cultured in the ELISpot plate for 20 h with 10^4^ of autologous PBMCs that were pulsed with 100 μM individual long peptide and irradiated (3000 Rad). The ELISpot plates were scanned and analyzed on an ImmunoSpot Reader (CTL, Shanker Heights, OH).

### Statistics

Data were analyzed using GraphPad Prism 8 software (GraphPad, La Jolla, CA) and presented mainly as mean ± SEM. The Mann-Whitney test or one-way ANOVA test were used to compare between vaccination groups. Paired *t*-test was performed in some cases when different conditions were compared using the same specimens. A *P* value equal or less than 0.05 is considered statistically significant. Figures were prepared using Adobe Illustrator CS6 (Adobe, San Jose, CA).

## Results

### Optimizing the design of polyepitope neoantigen DNA vaccines for enhanced presentation and recognition

**Fig. 1:**
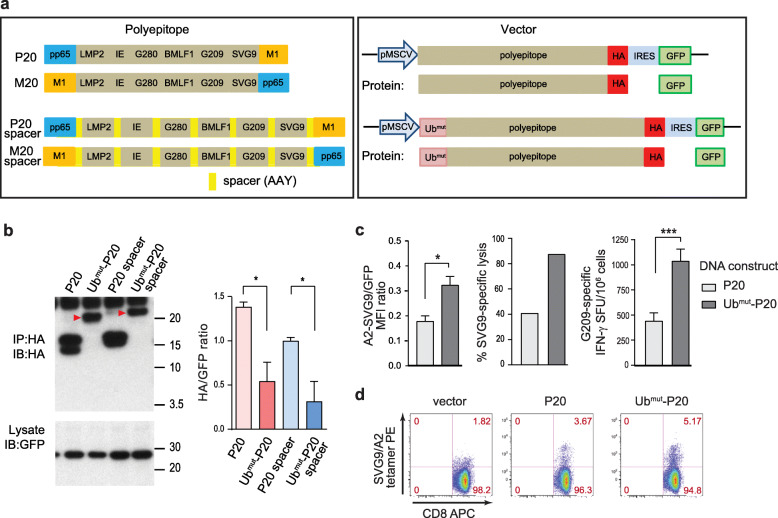
Optimizing the polyepitope DNA vaccine design. **a** Schematic DNA constructs encoding eight polyepitope model antigens (peptide sequences were listed in Additional file 2, Table S1). *Left*, polyepitope P20 and M20 differ only in the position of epitopes pp65 and M1. *Right*, the polyepitope constructs were subcloned into a retroviral vector driven by the MSCV promoter. The HA-tag and IRES-GFP were included to facilitate the in vitro detection of polyepitope protein production. Ub^mut^, a mutated (G76V) ubiquitin. **b** Immunoblot (IB) analysis of the polyepitope proteins. *Left*, HeLa-A2 cells were transduced with indicated polyepitope constructs. Red arrowheads indicate the ubiquitinated polyepitope proteins. *Right*, HA/GFP ratio was used to quantify relative levels of polyepitope proteins. Results combined from three independent experiments (mean ± SEM) were shown. **c** Presentation of antigens by the transduced HeLa-A2 cells. *Left*, surface staining of the SVG9/HLA-A2 complexes with a TCR-mimic antibody. Mean fluorescence intensity (MFI) of the SVG9/HLA-A2 signal relative to MFI of the co-expressed GFP (mean ± SEM, in triplicates) was shown. *Middle*, specific lysis of transduced HeLa-A2 cells by SVG9-specific cytotoxic T cells was measured by a ^51^Cr-releasing cytotoxicity assay (E:T = 25:1). *Right*, DNA vaccines induced G209-specific immune response in HHD II mice was measured by an IFN-γ ELISpot assay (mean ± SEM, *n* = 8). These experiments were repeated at least once and representative results were shown. **d** Representative dot plots showing SVG9/HLA-A2 tetramer staining of CD8^+^ spleen cells from the vaccinated HHD II mice. Numbers indicate frequencies in each quadrant. *P < 0.05, ***P < 0.001, *t*-test

We first established a model system to optimize the polyepitope DNA vaccine platform by using eight well-characterized HLA-A2-restricted epitopes. This model system allowed us to address important questions about polyepitope design such as size of the neoantigen epitope, inclusion of spacers, and addition of a ubiquitin tag to enhance antigen processing. The HLA-A2-restricted epitopes included viral (EBV, HCMV, influenza, and West Nile Virus) and tumor-associated antigens (melanoma gp100) (Additional file 2, Table S1). With the exception of the CMV (pp65) and influenza (M1) epitopes, the order of the other six epitopes was consistent between the model polyepitope constructs (Fig. 1a, *left*). The spacer inserted between epitopes consisted of three amino acids (AAY) [24]. To study whether antigen processing efficacy is different for short vs. long epitopes encoded in polyepitope DNA vaccines, we created polyepitope DNA constructs that encode either minimal epitopes (9–10 AA) or longer epitopes (20 AA) with native residues flanking the minimal epitopes. We designated the constructs as P9/P20 (starting with pp65) and M9/M20 (starting with M1). To facilitate in vitro assays, the polyepitope constructs integrated an HA tag at the C-terminus. Co-expression of GFP was made possible through an IRES element and served as control for transduction as measured in immunoblot (IB) analysis or flow cytometry (Fig. 1a, *right*).

To study the expression and processing of the polyepitope constructs, HeLa-A2 cells were transduced with different constructs. Cell lysates were immunoprecipitated (IP) followed by IB to detect HA-tagged polyepitopes. We found that GFP proteins were equally expressed in the transduced cells, as determined by IB. However, the P9 and P9-spacer constructs were undetectable while M9 and M9-spacer constructs were readily detected (Additional file 3, Fig. S1a, *left*), suggesting that polyepitopes starting with an unstable amino acid (such as N in pp65) degraded more rapidly than constructs starting with a stable residue (such as G in M1). This observation is consistent with the N-end rule of protein degradation [25]. The addition of spacers in both P9 and M9 constructs resulted in increased surface presentation of SVG9 as determined by flow cytometric analysis using a TCR-mimic antibody specific for SVG9/HLA-A2 [23]. But in the case of construct P20, the addition of spacers did not increase the presentation of SVG9 (Additional file 3, Fig. S1a, *middle and right*). These data suggest that additional amino acids in the spacers flanking the 9-mer epitopes might help with processing of the intact minimal epitope, but that spacers may not be required if native flanking sequences are present. We therefore decided to focus on the DNA construct P20, which encodes long epitopes.

Because MHC I binding peptides are initially processed in the cytosol by the ubiquitin/proteasome pathway [26], we hypothesized that integrating a mutant form of ubiquitin (Ub G76V or Ub^mut^), which is resistant to ubiquitin hydrolysis, to the N-terminus of the polyepitope constructs could accelerate ubiquitination-mediated degradation of the polyepitopes and antigen processing. Indeed, integration of Ub^mut^ before the polyepitope construct resulted in faster protein degradation, compared with the same construct without the addition of Ub^mut^, as determined by IB analysis (Fig. 1b). The integration of Ub^mut^ does not impact the transduction efficacy and protein expression, as the GFP levels were similar comparing constructs with or without the Ub^mut^ tag. Notably, this increased polyepitope degradation appeared to be associated with a higher surface presentation of HLA-A2/SVG9 complexes (Fig. 1c, *left*). The degradation of polyepitope proteins was proteasome-dependent, as addition of the proteasomal inhibitor MG132 protected the proteins from rapid degradation (Additional file 3, Fig. S1b). Consistent with the flow cytometric analysis, in a ^51^Cr-releasing cytotoxicity assay using an A2/G209-specific T cell line, HeLa-A2 cells transduced with Ub^mut^-P20 were lysed more efficiently compared to cells transduced with P20 (Fig. 1c, *middle;* Additional file 3, Fig. S1c). Additionally, when the DNA constructs were used to vaccinate HHD II mice, the Ub^mut^-P20 vaccine generated more robust CD8 T cell responses in vivo compared to the P20 vaccine, as determined by an IFN-γ ELISpot assay (Fig. 1c, *right;* Additional file 3, Fig. S1d) and SVG9/HLA-A2 tetramer staining (Fig. 1d).

Taken together, these results suggested that a polyepitope DNA construct encoding long epitopes (≥20 mers), in tandem with an Ub^mut^ fused to the N-terminus, is optimal for processing and presentation of epitopes among the constructs tested. This design was therefore used for subsequent studies. Of note, although the optimized vaccine is superior for the majority of the encoded antigens, it is not superior for all antigens compared to the control vaccine (Additional file 3, Fig. S1c).

### Optimized polyepitope neoantigen DNA vaccines elicit immune responses in preclinical mouse breast cancer models

**Fig. 2:**
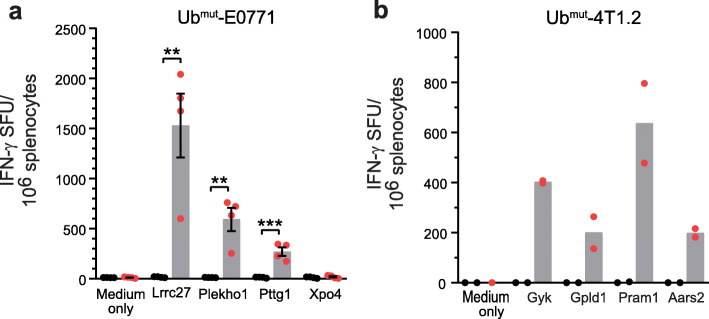
Polyepitope neoantigen DNA vaccine elicit neoantigen-specific T cell responses in vivo. Neoantigens were identified for E0771 and 4T1.2 breast cancer models. Polyepitope neoantigen DNA vaccines were created for each and were used to immunize mice by gene gun. Spleen cells from mice vaccinated with polyepitope DNA vaccines (red) and control empty vector DNA (black) were harvested and used in IFN-γ ELISpot assay. T cell responses to selected neoantigens were shown (mean ± SEM) for Ub^mut^-E0771 (**a**) and Ub^mut^-4T1.2 (**b**). Of note, 8- to 10-mer minimal peptides were used in the assays for Ub^mut^-E0771 (**a**), but 29-mer long peptides were used for Ub^mut^-4T1.2 (**b**). Experiments were repeated at least two more times for panel **a**, and similar results were obtained. **P < 0.01, ***P < 0.001, *t*-test

We carried out proof-of-concept studies using E0771 and 4T1.2, two syngeneic murine mammary tumors. E0771 and 4T1.2 recapitulate many of the biologic features of human breast cancer, including the dynamic tumor and immune system interactions restraining endogenous immune responses and serve as models of estrogen receptor-positive (ER^+^) and triple-negative breast cancer (TNBC), respectively [27, 28]. We sequenced both 4T1.2 and E0771 and successfully identified candidate neoantigens with strong predicted binding affinity to the corresponding MHC class I alleles using pVACseq, a computational pipeline [16, 17] (Additional file 2, Table S2 and S3). Polyepitope Ub^mut^-E0771 and Ub^mut^-4T1.2 neoantigen DNA vaccines were created and used to vaccinate C57BL/6 or Balb/c mice, respectively. Neoantigen-specific T cell responses were detected by IFN-γ ELISpot assay for three neoantigens (Lrrc27 G330A, Plekho1 P251S, and Pttg1 V53L) encoded in the Ub^mut^-E0771 polyepitope DNA vaccine (Fig. 2a). Of note, short peptides corresponding to the minimal MHC class I epitopes were used in these assays, suggesting a CD8 T cell response. Neoantigen-specific T cell responses were also detected by IFN-γ ELISpot assay for four neoantigens (Gyk K505R, Gpld1 R829W, Pram1 Q572L and Aars2 A697P) encoded in the Ub^mut^-4T1.2 polyepitope DNA vaccine (Fig. 2b). All four 4T1.2 neoantigens are known to contain CD4 epitopes as spleen CD4^+^ T cells isolated from mice vaccinated with peptides responded to peptide restimulation ex vivo (Additional file 3, Fig. S2). These results confirmed the ability of neoantigen DNA vaccines incorporating Ub^mut^-polyepitope inserts to generate robust immune responses in clinically relevant preclinical models.

We created additional constructs that did not integrate the mutant ubiquitin tag in the E0771 model system. E0771 polyepitope DNA vaccines with or without the Ub^mut^ tag were used to vaccinate C57BL/6 mice. Results from IFN-γ ELISpot assays demonstrated no statistically significant difference in the ability to induce neoantigen-specific immune responses (data not shown).

### Optimized polyepitope neoantigen DNA vaccines in combination with checkpoint blockade inhibit tumor growth in preclinical models

**Fig. 3:**
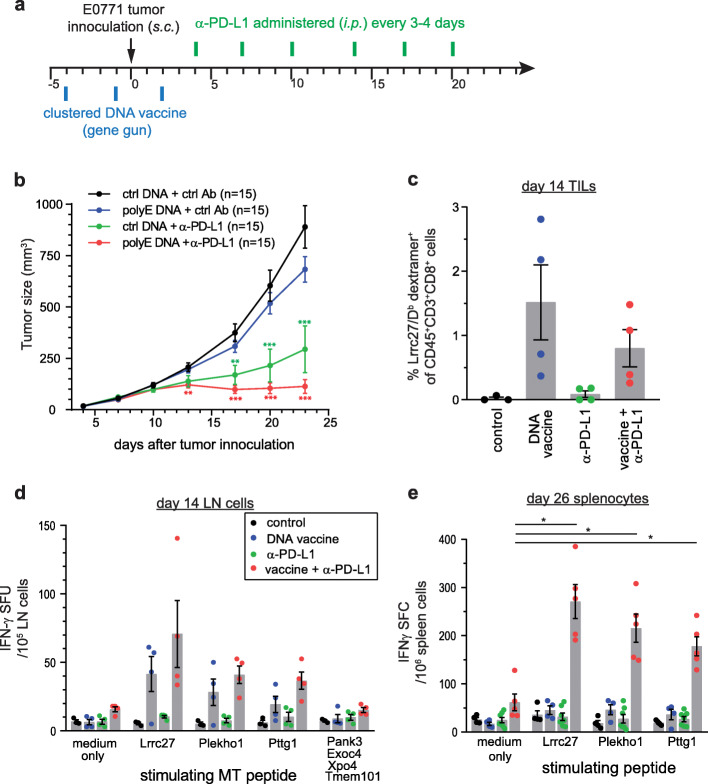
Polyepitope E0771 neoantigen DNA vaccines combined with anti-PD-L1 immunotherapy suppressed tumor growth in vivo. **a** Scheduling of DNA vaccination and anti-PD-L1 treatment. Wildtype female C57BL/6 mice (*n* = 15 per group) were vaccinated by gene gun on days − 4, − 1, and 2 and challenged with 10^6^ E0771 cells on day 0. Anti-PD-L1 or control antibodies were administered every 3–4 days. **b** Tumors were measured with electronic calipers of the longest (L) and perpendicular (W) diagonals. Tumor sizes (mean ± SEM) were calculated as *(L × W*^*2*^*)/2*. Results from one of the three independent experiments were shown. **c** In a parallel experiment, tumors were harvested and dissociated to prepare single cell suspension on day 14. TILs were analyzed by Lrrc27/D^b^ dextramer staining and flow cytometry. *P* = 0.0381, one-way ANOVA. **d** Tumor-draining lymph nodes (LN) were harvested on day 14. LN cells were used in an IFN-γ ELISpot assay and stimulated with selected MT peptides (8- to 10-mer). **e** Spleen cells were harvested from treated tumor-bearing mice on day 26 and used in an IFN-γ ELISpot assay. The studies were repeated once and similar results were obtained. Error bars, SEM. *P < 0.05, **P < 0.01, ***P < 0.001, *t*-test

We next investigated whether antitumor immunity can be generated by polyepitope neoantigen DNA vaccines. In an initial study, polyepitope Ub^mut^-E0771 DNA vaccine alone, in either prophylactic or therapeutic settings, had only a marginal impact on subcutaneously transplanted E0771 tumor growth (data not shown). However, when combined with anti-PD-L1 ICB therapy, the polyepitope Ub^mut^-E0771 neoantigen DNA vaccine was able to enhance the antitumor response and suppress E0771 tumor growth for the duration of the experiment (Fig. 3a, b). At day 14, robust neoantigen-specific T cell responses were detected in tumors (Fig. 3c) and tumor-draining lymph nodes (Fig. 3d) following treatment with neoantigen DNA vaccines alone, or neoantigen DNA vaccines plus anti-PD-L1 antibody. At day 26, neoantigen-specific T cell responses persisted only in mice treated with neoantigen DNA vaccines plus anti-PD-L1 antibody (Fig. 3e). In mice that received neoantigen DNA vaccines but not anti-PD-L1 antibody, neoantigen-specific T cell responses returned to baseline. These data suggest that in the setting of a tumor-bearing mouse, addition of anti-PD-L1 is required for persistent antitumor immunity following neoantigen DNA vaccine treatment.

Unlike E0771, which is responsive to anti-PD-L1 treatment, 4T1.2 is resistant to anti-PD-L1 monotherapy (Additional file 3, Fig. S3a). In a pilot study, we found that Ub^mut^-4T1.2 polyepitope DNA vaccine alone was able to partially inhibit tumor growth in vivo (Additional file 3, Fig. S3b). Further investigation is needed to understand the changes in immune system and whether ICB treatment will enhance the antitumor immunity induced by Ub^mut^-4T1.2 polyepitope DNA vaccine.

### Optimized polyepitope DNA vaccines induce similar magnitude of immune responses as synthetic long peptides

**Fig. 4:**
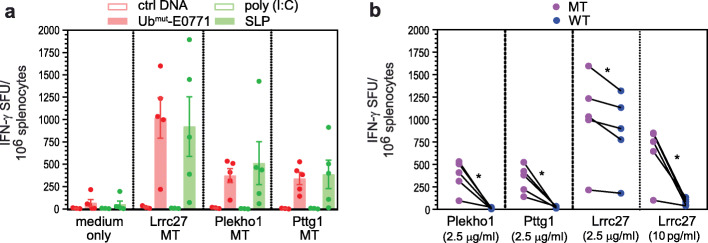
Polyepitope DNA vaccine generated similar magnitude of immune responses as synthetic long peptide vaccines. **a** Comparison of IFN-γ ELISpot results (mean ± SEM) induced by polyepitope Ub^mut^-E0771 DNA vaccine and SLP vaccine. Wildtype C57BL/6 mice were vaccinated with Ub^mut^-E0771 vaccine or a mixture of three SLPs. The schedule for both platforms was optimized independently. The IFN-γ ELISpot assay was performed on the same day when immune responses are at peak level. The experiment was repeated once and similar results were obtained. **b** Specificity of DNA vaccine-generated immune response towards neoantigens (MT) over corresponding WT peptides. An IFN-γ ELISpot assay was performed by using 8- to 10-mer MT and WT peptides at different concentrations. Results shown were from one of the two independent experiments. Results generated with high (2.5 μg/ml) and low (10 pg/ml) MT/WT Lrrc27 peptides were also shown. *P < 0.05, paired *t*-test

We compared the efficacy of polyepitope neoantigen DNA vaccines with that of neoantigen SLP vaccines. Vaccine schedules were optimized for each platform by testing different doses and vaccination time points (DNA vaccine), or different doses, vaccination time points and molecular adjuvants (SLP vaccine) (data not shown). IFN-γ ELISpot assays performed on the same day indicated that the Ub^mut^-E0771 polyepitope neoantigen DNA vaccine and the neoantigen SLP vaccine generated similar levels of T cell responses specific to the three neoantigens (Fig. 4a). Likewise, polyepitope Ub^mut^-4T1.2 DNA vaccine and SLP vaccine generated similar levels of T cell responses specific to the four neoantigens (data not shown).

Specificity of the immune response was further investigated by means of cross-reactivity against corresponding germline (wildtype) Lrrc27, Plekho1, and Pttg1 epitopes over a range of concentrations. At physiologic concentrations, no reactivity was detected against all three WT epitopes (Fig. 4b and data not shown). Of note, one of the wild-type peptides (Lrrc27) was predicted to be a strong binder, with a predicted binding affinity of 408.48 nM to H-2D^b^ [netMHC 4.0 [29], http://www.cbs.dtu.dk/services/NetMHC]. Additional analysis revealed that both MT and WT Lrrc27 peptides bind equally well to H-2D^b^ (Additional file 3, Fig. S4A). At relatively high concentration, some cross-reactivity was observed against WT Lrrc27 peptide following vaccination with either polyepitope DNA or SLP vaccines (Fig. 4b and Additional file 3,Fig. S4b). These data suggest that T cells induced by polyepitope Ub^mut^-E0771 DNA vaccines preferably recognize MT neoantigens over WT counterparts when the density of peptide-MHC complexes is low. Such specificity to neoepitopes is critical as tumor cells typically only display relatively few neoantigen-MHC complexes.

### An optimized polyepitope neoantigen DNA vaccine induced neoantigen-specific T cell responses in a patient with metastatic neuroendocrine tumor

**Fig. 5:**
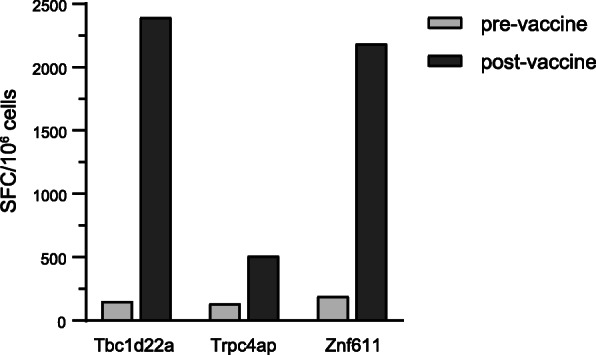
An optimized polyepitope neoantigen DNA vaccine is capable of inducing neoantigen-specific T cell responses in a patient with metastatic pancreatic neuroendocrine cancer. PBMC from patient GTB16 were obtained before (pre-vaccine) and after (post-vaccine) vaccination with an optimized polyepitope neoantigen DNA vaccine. PBMC were stimulated in vitro for 12 days with peptides corresponding to the indicated neoantigens and then an IFNγ ELISpot assay was performed. The number of spot forming cells (SFC) specific for each neoantigen is indicated. Nonspecific background counts, assessed by incubating cells without peptide during the ELISpot assay, were subtracted. The assays were repeated twice and similar results were obtained. Please note that the vaccine incorporated 13 neoantigens. A robust response was observed to 3/13 neoantigens. The other neoantigens did not induce a response

Cancer patient GTB16 was treated with an optimized polyepitope neoantigen DNA vaccine. The pGTB16 vaccine was constructed as described for the preclinical studies and was manufactured in the GMP facility at WUSM. The DNA sequence of the pGTB16 construct and a list of targeted neoantigens can be found in Additional file 1 and Additional file 2, Table S4. IFNγ ELISpot assay performed after in vitro stimulation indicated that the polyepitope neoantigen DNA vaccine was able to induce T cell responses against select neoantigens. For this patient, 13 neoantigens were targeted by the DNA vaccine. Specific responses above background were demonstrated against three neoantigens (*TBC1D22A*:p.R437S, *TRPC4AP*:p.T63M, and *ZNF611*:p.D404G, Fig. 5).

## Discussion

Recombinant DNA vaccines can generate potent immune responses [30–33] and have progressed into clinical trials targeting infectious disease agents and cancer [34]. DNA vaccines are stable, relatively easy to design and manufacture, and less expensive than synthetic long peptide, viral or cell-based vaccine platforms. More importantly, the molecular flexibility of the DNA vaccine platform allows genetic modification of encoded antigens, and/or incorporation of immune modulators to improve immunogenicity. In this study, we constructed polyepitope DNA vaccines encoding multiple cancer neoantigens and evaluated multiple parameters of the vaccine design including the length of neoepitopes, inclusion of spacers, and/or inclusion of a mutant ubiquitin construct to enhance antigen presentation.

The polyepitope approach has been studied previously and proved to be effective in priming T cell responses against viral and conventional tumor antigens [24, 35–37]. Polyepitope proteins require additional intracellular processing in order to be loaded onto MHC molecules. Studies of peptide vaccination in incomplete Freund’s adjuvant (IFA) showed that longer peptides (25-mers) in IFA can generate sustained CD8 T cell reactivity while shorter peptides (8–10-mers) corresponding to minimal epitopes induced only short-lived CD8 T cell responses [38]. This may result from altered antigen processing with minimal epitopes, or the inclusion of both CD4 and CD8 epitopes in the longer epitopes. Since the goal of neoantigen cancer vaccines is to generate a robust and long-lasting cancer-specific immune response, we chose to evaluate both short and long neoepitopes in the DNA vaccines, with the understanding that maximizing presentation of long neoepitopes would be preferred. There is now evidence to suggest that long neoantigens may contain neoantigen-specific CD4 helper T cell epitopes with the potential to induce a more balanced CD8/CD4 response. Recent studies demonstrate that CD4 T cell responses were induced by neoantigen vaccines even though neoantigen prioritization was based primarily on the binding affinity to MHC class I [7, 9, 39]. Our data confirm that long neoantigens are equally well processed and can induce robust neoantigen-specific CD8 and CD4 T cell responses in vivo.

Amino acids flanking minimal epitopes play an important role in TAP-binding and proteasomal cleavage. Researchers have used artificial linkers [24, 37] and furin cleavage sites [36] to facilitate proteolytic cleavage and antigen presentation. Previous studies indicated a preference of natural flanking sequences in TAP-dependent antigen presentation [40]. Some peptides are efficiently presented by MHC I molecules but are poorly transported by TAP as minimal epitopes. Research suggested that they can be more efficiently transported by TAP as larger fragments with natural flanking amino acids, which can be further trimmed in the endoplasmic reticulum (ER) and bind to MHC class I molecules [40]. In the current study, we found that epitopes flanked by natural sequences can be processed and presented effectively and adding a linker does not further enhance antigen presentation. In order to minimize the risk of junctional neoepitopes, we have developed a robust tool (pVACvector) to assess for the presence of junctional epitopes. pVACvector starts with the list of prioritized neoantigens and uses the pVACseq software to predict the binding score for each possible junctional peptide. This information is used to order the neoantigen sequences in a way that minimizes junctional epitopes [17]. Current version of the pVACtools suite, which contains pVACseq and pVACvector, is available at Github [41].

To enhance antigen presentation, we explored integration of a mutant ubiquitin tag as part of the polyepitope DNA vaccine to facilitate protein degradation and maximize antigen presentation. Early studies in the yeast *Saccharomyces cerevisiae* demonstrated that a monoubiquitin conjugate can function as protein degradation signal [42]. Fusion of a ubiquitin molecule to a polyepitope DNA vaccine was able to enhance CTL priming and improve antitumor immune responses in an HPV-induced preclinical model [24]. However, natural ubiquitin fusions are unstable and prone to deubiquitination under physiological conditions. The modification at the C-terminal portion of ubiquitin, replacing the glycine with a valine (G76V), metabolically stabilizes the fusion as revealed by pulse-chase analysis [42, 43]. This “uncleavable” Ub^mut^ has been a useful tool in studying cell cycle and apoptosis [18], as wells as autophagy [44]. We have demonstrated in this study that the Ub^mut^ tag does indeed improve antigen processing and presentation, which in turn results in an enhanced immune response in vitro (Fig. 1).

We created polyepitope DNA vaccines encoding neoantigens identified in mouse breast cancer models and tested these vaccines in vivo. Polyepitope DNA vaccines were able to induce robust T cell responses to some but not all neoantigens. This underscores the need for further improvement of neoantigen prediction algorithms. Our finding that a polyepitope neoantigen DNA vaccine can induce robust T cell responses and antitumor immunity is consistent with a recent report [36]. Although our findings confirm and extend the results of Duperret et al., the polyepitope DNA vaccine designs evaluated here are distinct. Duperret et al. constructed polyepitope DNA vaccines encoding 33-mer neoantigens separated by furin cleavage sites. We demonstrate that furin cleavage sites are not required for robust neoantigen presentation, and we have leveraged a publically available software tool, pVACvector, to optimize the order of neoantigens so that the risk of introducing junctional epitopes is minimized. In addition, we integrated the Ub^mut^ tag to the constructs, which clearly improves antigen processing and presentation, and potentially the downstream immune responses. Of note, the optimized polyepitope vaccine is not superior for every neoantigen/model tested. For instance, a side-by-side comparison of polyepitope DNA vaccines with or without the Ub^mut^ tag targeting the same neoantigens identified in E0771 showed no statistically significant difference in magnitude of neoantigen-specific responses. We hypothesize that some neoantigens are efficiently processed and may not require targeting to the ubiquitin pathway for presentation, while others are less efficiently processed and benefit from targeting to the ubiquitin pathway.

In spite of the ability to generate neoantigen-specific T cell responses, the neoantigen DNA vaccines alone were not able to protect animals from transplanted E0771 and 4T1.2 tumors. We present our observation that combinatorial immunotherapy of neoantigen DNA vaccine plus anti-PD-L1 checkpoint blockade is capable of suppressing E0771 tumor growth. One limitation in our study is that we did not study in detail the mechanism(s) of the antitumor immunity rendered by the optimized polyepitope neoantigen DNA vaccine +/− ICB immunotherapy. Research into the cellular and molecular changes occurring in the tumor microenvironment following combination therapy with ICB and neoantigen DNA vaccine is currently underway in novel genetic models. Preliminary data suggest that tumor growth inhibition by combination immunotherapy is associated with sustained neoantigen-specific T cell responses and CD8 T cell infiltration into the tumor (Fig. 3c–e). The recent clinical success of ICB in treating various types of cancer [reviewed in [45–47]] has pushed it towards the forefront of cancer therapy. We have initiated a randomized phase 1 clinical trial that tests the efficacy of a polyepitope neoantigen DNA vaccine +/− anti-PD-L1 in patients with triple negative breast cancer (NCT03199040). This trial represents unique bench-to-bedside-to-bench opportunities to enhance the efficacy of neoantigen vaccines and checkpoint blockade therapy.

Finally, we have treated a patient with metastatic neuroendocrine tumor with a polyepitope neoantigen DNA vaccine. This is the first report of the use of a neoantigen DNA vaccine in humans. The tumor of this patient was refractory to standard of care treatment and showed evidence of ongoing progression at the time when the vaccination started on a compassionate use basis. Our data demonstrate that polyepitope neoantigen DNA vaccines are capable of inducing neoantigen-specific T cell responses. The successful clinical translation of polyepitope neoantigen DNA vaccines will likely depend on refinement of neoantigen prediction algorithms, combination therapies targeting the tumor microenvironment, and an improved ability to assess the antitumor potential of neoantigen-specific T cells. In this study, we observed neoantigen-specific T cell responses to 3/13 neoantigens included in the vaccine, highlighting the need to further refine current neoantigen prediction algorithms. In addition, neoantigen-specific T cells may not be effective in mediating antitumor immunity if these T cells are suppressed in the tumor microenvironment. For future studies, we are planning to use innovative technologies such as CyTOF, IMC, and CODEX, to investigate the phenotype and function of neoantigen-specific T cells in the tumor, and the impact of combination therapy on the tumor microenvironment. These technologies will allow a better understanding of the antitumor potential of neoantigen-specific T cells.

## Conclusions

We have optimized a polyepitope DNA vaccine design to encode multiple neoantigens. Tumor/normal whole exome sequencing and RNA sequencing were used to identify and prioritize neoantigens in the E0771 and 4T1.2 preclinical breast cancer models, as well as a patient with metastatic pancreatic neuroendocrine tumor. E0771 and 4T1.2-specific polyepitope neoantigen DNA vaccines were able to induce robust immune responses and inhibit tumor growth when combined with anti-PD-L1 checkpoint blockade immunotherapy. Similarly, neoantigen-specific immune responses were detected after vaccination in a patient with metastatic neuroendocrine tumor. The results provide strong evidence to support clinical translation of a polyepitope neoantigen DNA vaccine strategy. We are currently evaluating the polyepitope neoantigen DNA vaccine platform in phase 1 clinical trials in breast and pancreas cancer (NCT03199040 and NCT03122106).

## Supplementary Information

**Additional file 1.** This file contains the DNA and amino acid sequences of the polyepitope DNA vaccines created for this study.

**Additional file 2:** This file contains Supplementary Table S1 to S4. **Table S1.** List of HLA-A2-restricted epitopes included in the octamers for optimizing polyepitope DNA vaccine design; **Table S2.** Selective neoantigens identified in E0771 mouse breast cancer; **Table S3.** Neoantigens identified in mouse breast cancer cell line 4T1.2; and **Table S4.** Neoantigens identified in cancer patient GTB16.

**Additional file 3:** This file contains Supplementary Methods and Supplementary Figure S1 to S4. **Figure S1.** Expression of polyepitope constructs and the presentation of antigens. **Figure S2.** SLP vaccines generated neoepitope-specific CD4 T cell responses. **Figure S3.** Ub^mut^-4T1.2 Polyepitope neoantigen DNA vaccine inhibits 4T1.2 tumor growth in vivo*.* **Figure S4.** Both MT and WT Lrrc27 peptides bind well to H-2D^b^.

## Data Availability

Processed data generated during this study are included in this published article and its additional files. Mouse sequence data are available at the Sequence Read Archive under BioProject PRJNA685845 https://www.ncbi.nlm.nih.gov/bioproject/?term=PRJNA685845 [48]. Human sequence data/analyses have been deposited in and are available from the dbGaP database under dbGaP study accession phs002342.v1.p1 http://www.ncbi.nlm.nih.gov/projects/gap/cgi-bin/study.cgi?study_id=phs002342.v1.p1 [49].

## Abbreviations

CTL: Cytotoxic T lymphocyte
DC: Dendritic cell
ELISpot: Enzyme-linked immune absorbent spot
ER: Endoplasmic reticulum
HLA: Human leukocyte antigen
ICB: Immune checkpoint blockade
IFA: Incomplete Freund’s adjuvant
IP/IB: Immunoprecipitation/immunoblot
MHC: Major histocompatibility complex
MT/WT: Mutated/wildtype
PBMC: Peripheral blood mononuclear cell
SLP: Synthetic long peptide
TAP: Transporter associated with antigen processing
TCR: T cell receptor
TIL: Tumor-infiltrating leukocyte
TNBC: Triple-negative breast cancer
