# Supplementary material for: Optimized polyepitope neoantigen DNA vaccines elicit neoantigen-specific immune responses in preclinical models and in clinical translation
Source: Genome Med. 2021 Apr 21;13:56. doi: 10.1186/s13073-021-00872-4 (PMC8059244; doi:10.1186/s13073-021-00872-4)
Supplement: Supplementary file 1 — Additional file 1. This file contains the DNA and amino acid sequences of the polyepitope DNA vaccines created for this study. [file 13073_2021_872_MOESM1_ESM.docx]

**Polyepitope DNA vaccine sequences**

Listed below are the DNA sequences (blue letters) and translated amino acid (AA) sequences (black letters) of Ub^mut^ and polyepitope vaccine constructs. Of note, when Ub^mut^ was subsequently cloned in front of the polyepitope constructs, the start codon (ATG) and the encoded methionine (M) were removed from each construct, respectively.

For the DNA constructs that encode HLA-A2 epitopes, only P20 containing a C-terminus HA tag (YPYDVPDAY) is listed. Sequences for the related constructs M20, P9, and M9 with or without the AAY spacer are available upon request. DNA constructs were cloned into pMSV.IRES.GFP (*in vitro* studies) or pcDNA3.1^(+)^ (*in vivo* studies) under the control of a CMV promoter. Personalized DNA vaccine construct for the cancer patient GTB16 was cloned into pING vector (1). In 4T1.2 polyepitope DNA vaccine, sequence encoding a murine CMV pM84 peptide which contains the H-2K^d^-restricted epitope AYAGLFTPL (2) was included as a built-in positive control.

* denotes the translational stop.

Reference

1. Bergman PJ, McKnight J, Novosad A, Charney S, Farrelly J, Craft D, et al. Long-term survival of dogs with advanced malignant melanoma after DNA vaccination with xenogeneic human tyrosinase: a phase I trial. Clin Cancer Res. 2003;9(4):1284-90.

2. Holtappels R, Thomas D, Reddehase MJ. Identification of a K(d)-restricted antigenic peptide encoded by murine cytomegalovirus early gene M84. J Gen Virol. 2000;81(Pt 12):3037-42.

**Ub^mut^ (G76V)**

*DNA (5’🡪3’)*

atgcagatctttgtgaaaaccttaactggtaagaccatcaccctggaggtcgagcccagtgacaccattgagaatgtcaaggcaaagatccaggacaaggagggcatcccccctgaccagcagaggctgatctttgcaggcaagcagctggaagatggccgcaccctgtcagactacaacatccagaaagagtccaccctgcacctggtccttcgcctcagaggtgtc

*AA (N🡪C terminus)*

MQIFVKTLTGKTITLEVEPSDTIENVKAKIQDKEGIPPDQQRLIFAGKQLEDGRTLSDYNIQKESTLHLVLRLRG**V**

**P20-HA polyepitope vaccine**

*DNA (5’🡪3’)*

ggcatcctggccagaaacctggtgcccatggtggccaccgtgcagggccagaacctgaagggccccgtgttcatgtgcctgggcggcctgctgaccatggtggccggcgccgtgtggctggtgctgtgctgctacgtgctggaggagaccagcgtgatgctggccaagagacccctgatcgtggtgacccacacctacctggagcccggccccgtgaccgcccaggtggtgctgcaggccgccatccagaacgccggcctgtgcaccctggtggccatgctgctggaggagaccatcttcagcagcgccttcaccatcaccgaccaggtgcccttcagcgtgcagctgagagccctggacgcctgggacttcggcagcgtgggcggcgtgttcaccagcgtgggcaaggccgtgcaccagagccccctgaccggcggcatcctgggcttcgtgttcaccctgaccgtgcccagcgagagacaattgtacccatacgatgttccagattacgcttag

*AA (N🡪C terminus)*

GILARNLVPMVATVQGQNLKGPVFMCLGGLLTMVAGAVWLVLCCYVLEETSVMLAKRPLIVVTHTYLEPGPVTAQVVLQAAIQNAGLCTLVAMLLEETIFSSAFTITDQVPFSVQLRALDAWDFGSVGGVFTSVGKAVHQSPLTGGILGFVFTLTVPSER QLYPYDVPDYA*

**E0771 polyepitope vaccine**

*DNA (5’🡪3’)*

atgcagctggcctctacctacacagcttacatcgtgggctacgtgcactacggagattggctgaagaacgagaagatcaacagggtggtgttcgtgtggaactttctgcgggtgaacaccctgtctatgcgcccttgggagaagccagacaagggcgccagctacacaccacaggctctgaagaagttccccgccgacctggattttgctagacagtactacgtgatgctgtacaacaccgccgacgagctgctgttcaagggaatcctgcccaacctgcctagcgcctaccagaacaccgtgcacgctaacaggatgacagattctgtgatcagactgctgagcgccctgctgcgggtgtccgaggtggagtctagggctagagtgggcaaggtgtttaacgccccagctctgcccaaggcctccagaaaggctctgggcaccctgggaaaggagctgttcatgtactttggacaccgggccctgcgcatccacttcggaatgaagctgtttgaggacacaaacctgtgcgccatcaacgctaagcgggtgaccatcatgcctaaggacgacgtgagcgtgacaaaggccctgcagcacctgtctcactacttcgagggcgtgctgaagtgtctggtgagcatgccactgtgggctaagcacatgtccgatgagcagatccagggcttcgtggaggagccctttgagaagcctgtgatgatctctatgggaaacgagaacgtggtggagatcaagggcctggagatccagggaaccgaccctgtgagcgccgtgaccctgagcctgctggaccccgagacctga

*AA (N🡪C terminus)*

MQLASTYTAYIVGYVHYGDWLKNEKINRVVFVWNFLRVNTLSMRPWEKPDKGASYTPQALKKFPADLDFARQYYVMLYNTADELLFKGILPNLPSAYQNTVHANRMTDSVIRLLSALLRVSEVESRARVGKVFNAPALPKASRKALGTLGKELFMYFGHRALRIHFGMKLFEDTNLCAINAKRVTIMPKDDVSVTKALQHLSHYFEGVLKCLVSMPLWAKHMSDEQIQGFVEEPFEKPVMISMGNENVVEIKGLEIQGTDPVSAVTLSLLDPET*

**4T1.2 polyepitope vaccine**

*DNA (5’🡪3’)*

atgatttgcgatctgcttcagctgtttattggcgcacgccttgaatctgattaaattagtacgcggccgcaaaccgctgtcttggcttagccgcgccccgcgtccgaccggccctccagcgtcccgcttagcgagtaagagccacagcagcgttaaacgcctgcgtaaaatgcatcatgccgccgtgggacaggataaaccggtctttatggaggaagtcccccttccgcacacagcccgcattccgggactccggtcattggatgaagtccaaccgcagcagctgccagcaacagatccaaaactgctgaaattgatccgtaaggctgaaaaagccgaacgtgaatttcgtaaaaaagcggcgctgttggaaaccccacggggtaaaattcaggccaaaaaatggagcctggtaccgttttctattccggttttcgatattttgcaagattgcgcggccttgatcccgcacaatccacgcgtagcggtcaaaacgacgaacaacctcgttatgaaaaacagtgtttgcctggagcgcgattcgtttaccctgacggccctgcgtcgccgtggttttccgcctgacgccatcaacaacttctgcgcccgggtaggcgtaaccgtagctgctttaatggcgatggagctcttccgcgtctgtttggttgtggttacaggaattatcaatcaccctttgctgttcccgcgtgaaaacgcgacccaccactctaagatggacttagaaaaaccgaactacatcgtgccagattgtatgccggttgtttatgataagctgccacaaccgccgactcatcaccacggtcgtaaccaggttgtagtggctgcgggtcgtagtagctggggtgcttggctgagtggcgcgctccacgtgtatagcttttcaagtcatcatctgcgcgttgaaaaactgcaactcgagagcgagctgaacgaaagccgtacggaatgcatcaccgccacgtcgcagatgacggcccaccaccatgattctgttaccaagttcaaactgcaaggctcgccggtcccgcgccttcgtcagtccttattgtggggtgaaccggcacgtccgccgcatcatattaacgccgaggaatcggaaattcgctacagtacctggaagcgcgcggtaatgaagagcattggctgggttaccacacagagtccagttagtatcagctacttttcccgtgcggcgtacgcggggttgtttaccccgttataccgtccgggcctgtcatgccagatttaa

*AA (N🡪C terminus)*

MFAICFSCLLAHALNLIKLVRGRKPLSWLSRAPRPTGPPASRLASKSHSSVKRLRKMHHAAVGQDKPVFMEEVPLPHTARIPGLRSLDEVQPQQLPATDPKLLKLIRKAEKAEREFRKKAALLETPRGKIQAKKWSLVPFSIPVFDILQDCAALIPHNPRVAVKTTNNLVMKNSVCLERDSFTLTALRRRGFPPDAINNFCARVGVTVAALMAMELFRVCLVVVTGIINHPLLFPRENATHHSKMDLEKPNYIVPDCMPVVYDKLPQPPTHHHGRNQVVVAAGRSSWGAWLSGALHVYSFSSHHLRVEKLQLESELNESRTECITATSQMTAHHHDSVTKFKLQGSPVPRLRQSLLWGEPARPPHHINAEESEIRYSTWKRAVMKSIGWVTTQSPVSISYFSRAAYAGLFTPLYRPGLSCQI*

**pGTB16 polyepitope vaccine**

*DNA (5’🡪3’)*

ggcgacaactttcgtgagaccctgaaaaagaagaaacgcacgcttgtaatgttttacgcaccatggtgcccacaccatcatctgttaatgcgcgaagtgccactgcgttgtacgatcagcctttgggacacctaccagagcgaaccagacggtttccaccacactcgtcgcctgcgcgaacaacagaaaaccgcagaatgtgatgtgggcgattaccgttgtccgcaggatcagtctgcggcgttgctggtgcgcgctgtgcagttcacagaaacctttctgatggaacgcgataagcagagtaaatggtctggaatccctgcggcgtacgaagttttagaacaggagaaaggtgcactgtctgatggtgaaattgtgagtctgtctattgaattttacgaaggccatcactgcccgtcacctcaggcagagaaacgcttgccgaaattacacttagaaattatcgataaggactctaagacccgccgcgttaaaactgacagcaccggaacgcattcactctatgctatgtatcaggattacgaaattatgtttcatgtttcgcaccattgcgcccttggagcagccgcgctggcagtagtcaaaagcacgctggagtgggcgccgaaattccaactgcaactgttccaccacgccgcagtaacgaaagagcctattccggtcctgccgaccgttcgttataatatgggtggcatcccgactaattacaagggcaataccctggaacaggagcaggaagcgctggtgaatcacttgtggaagcgtatggataaattggaagcagagaaacaccatcattgtccgagtgcttattatgaggcagctctgctgcagctgtgggtcacagaagcgtgcacctaccgcccgtcagcacagcaccatcatgaccgttttccactgcgtaatgcagaaatggctaaagtcctggaaatttccagcgtgcctgcttcagaccgtatggtgcaccatcacaccggagaaaaaccatatcgttgcaaggtttgcgggaccgccttcacgtggcattcacagctggcccgccactaa

*AA (N🡪C terminus)*

GDNFRETLKKKKRTLVMFYAPWCPHHHLLMREVPLRCTISLWDTYQSEPDGFHHTRRLREQQKTAECDVGDYRCPQDQSAALLVRAVQFTETFLMERDKQSKWSGIPAAYEVLEQEKGALSDGEIVSLSIEFYEGHHCPSPQAEKRLPKLHLEIIDKDSKTRRVKTDSTGTHSLYAMYQDYEIMFHVSHHCALGAAALAVVKSTLEWAPKFQLQLFHHAAVTKEPIPVLPTVRYNMGGIPTNYKGNTLEQEQEALVNHLWKRMDKLEAEKHHHCPSAYYEAALLQLWVTEACTYRPSAQHHHDRFPLRNAEMAKVLEISSVPASDRMVHHHTGEKPYRCKVCGTAFTWHSQLARH*
