# Supplementary material for: Optimized polyepitope neoantigen DNA vaccines elicit neoantigen-specific immune responses in preclinical models and in clinical translation
Source: Genome Med. 2021 Apr 21;13:56. doi: 10.1186/s13073-021-00872-4 (PMC8059244; doi:10.1186/s13073-021-00872-4)
Supplement: Supplementary file 3 — Additional file 3: This file contains Supplementary Methods and Supplementary Figure S1 to S4. Figure S1. Expression of polyepitope constructs and the presentation of antigens. Figure S2. SLP vaccines generated neoepitope-specific CD4 T cell responses. Figure S3. Ubmut-4T1.2 Polyepitope neoantigen DNA vaccine inhibits 4T1.2 tumor growth in vivo. Figure S4. Both MT and WT Lrrc27 peptides bind well to H-2Db. [file 13073_2021_872_MOESM3_ESM.pdf]

Supplementary Methods

Peptide binding assay

To measure the binding affinity of MT and WT Lrrc27 peptides to H-2D^b molecules, RMA-S cells were pulsed with different concentrations of peptides and incubated overnight at 37°C. Cells were then stained with H-2D^d-specific monoclonal antibodies (clone 34-5-8) followed by PE-labeled secondary antibody (BD Biosciences). Monoclonal antibody was provided by Dr. T. Hansen (Washington University School of Medicine) as pre-titrated hybridoma supernatant. Mean Fluorescence index (MFI) was determined on a FACSCalibur flow cytometer (BD Biosciences).

CD4$^+$ T cell purification and ELISpot assay

Spleens were harvested from Balb/c mice vaccinated with SLPs. CD4$^+$ T cells were purified using the EasySep mouse CD4$^+$ T cell isolation kit (STEMCELL Technologies, Cambridge, MA) following manufacturer's instruction. Purified CD4$^+$ T cells were used in a modified IFN-γ ELISpot assay in which 10^5 of irradiated (3000 Rad) naïve spleen cells were added to each well with or without 5 μg/ml of each 29-mer neopeptides.

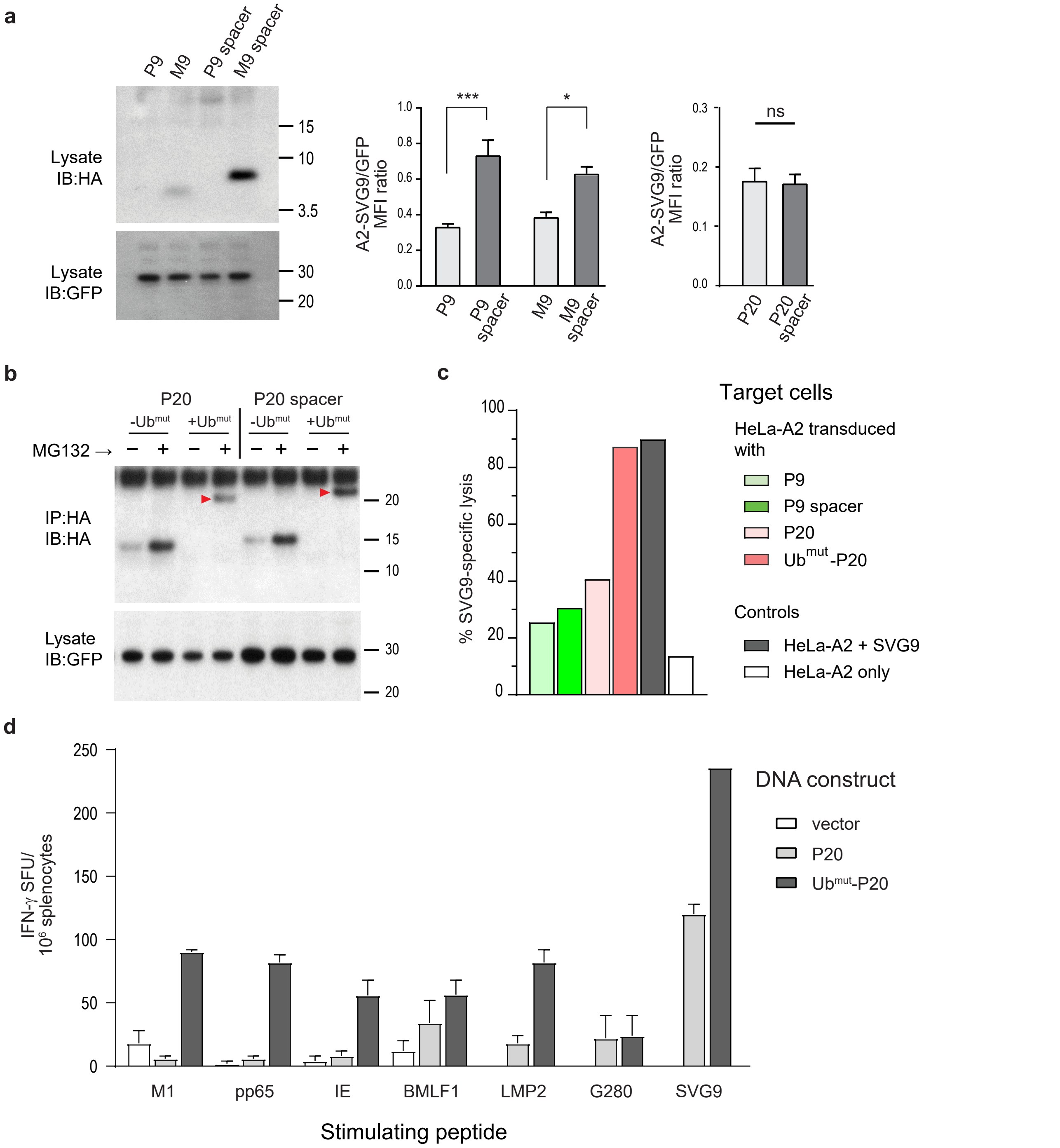

Fig S1. Expression of polyepitope constructs and the presentation of antigens. a *Left*, IB results showing expression and degradation of polyepitope antigens. *Middle* and *Right*, surface expression of WNV SVG9 on HeLa-A2 cells transduced with indicated polyepitope DNA constructs were measured by staining with a TCR-mimic antibody. The expression level was normalized to co-expressed GFP as A2-SVG9/GFP MFI ratio (mean ± SEM). **b** HeLa-A2 cells transduced with indicated polyepitope constructs were cultured for 2 hours with or without 50 μM of MG132 before subjected to IP. Red arrowheads indicate ubiquitinated polyepitope proteins. **c** Specific lysis of transduced HeLa-A2 by SVG9-specific T cells as measured by a ^{51}Cr-releasing cytotoxicity assay (E:T = 25:1). Target HeLa-A2 cells were transduced with indicated polyepitope constructs. Parental HeLa-A2 cells pulsed with or without SVG9 peptide were used as control. **d** DNA vaccines induced immune responses against model antigens in HHD II mice was measured by an IFN-γ ELISpot assay (mean ± SEM). The *in vitro* studies (**a-c**) were repeated at least twice and the *in vivo* experiment (**d**) was repeated once. Similar results were obtained. * $P < 0.05$, *** $P < 0.001$, *t*-test.

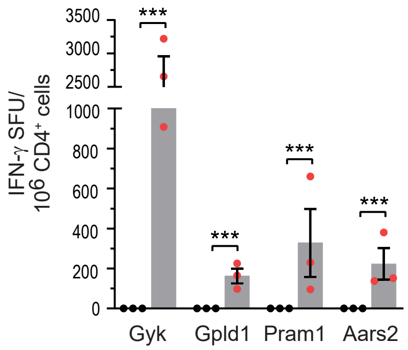

Fig S2. SLP vaccines generated neoepitope-specific CD4 T cell responses. Female Balb/c mice were vaccinated with individual SLP/poly(I:C) as described in the Methods. Spleen cells were harvested and purified $CD4^+$ T cells were used in an IFNγ ELISpot assay. Irradiated spleen cells from naïve mice were added into each well as feeder cells with (red) or without (black) corresponding long peptides. The experiment was repeated once and similar result was obtained. Error bars, SEM. *** $P < 0.001$, *t*-test.

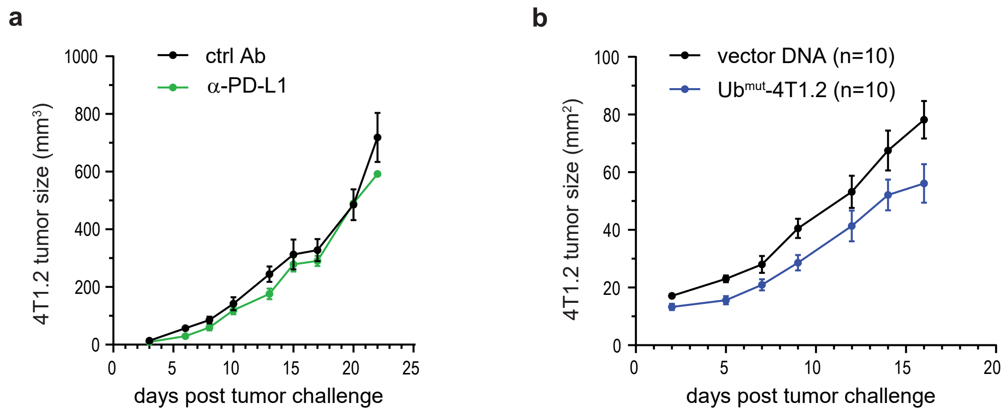

Fig S3. Ub^{mut}-4T1.2 Polyepitope neoantigen DNA vaccine inhibited tumor growth *in vivo*. a 4T1.2 is resistant to anti-PD-L1 monotherapy. 5×10^5 4T1.2 cells were injected subcutaneously into flanks of Balb/c mice. Starting on day 4, mice were treated with 200μg of anti-PD-L1 or isotype control antibody (*i.p.*) every three days for a total of three doses. Tumor sizes were measured with an electronic caliper and calculated as (L×W^2)/2. **b** Balb/c mice were vaccinated by gene gun with Ub^{mut}-4T1.2 or control vector DNA vaccine. Four days following the complete of DNA vaccination, mice were challenged with 10^6 4T1.2 cells subcutaneously in the flanks. Tumor sizes were measure with an electronic caliper and calculated as L×W. Error bars, SEM. The experiments were repeated once and similar results were obtained.

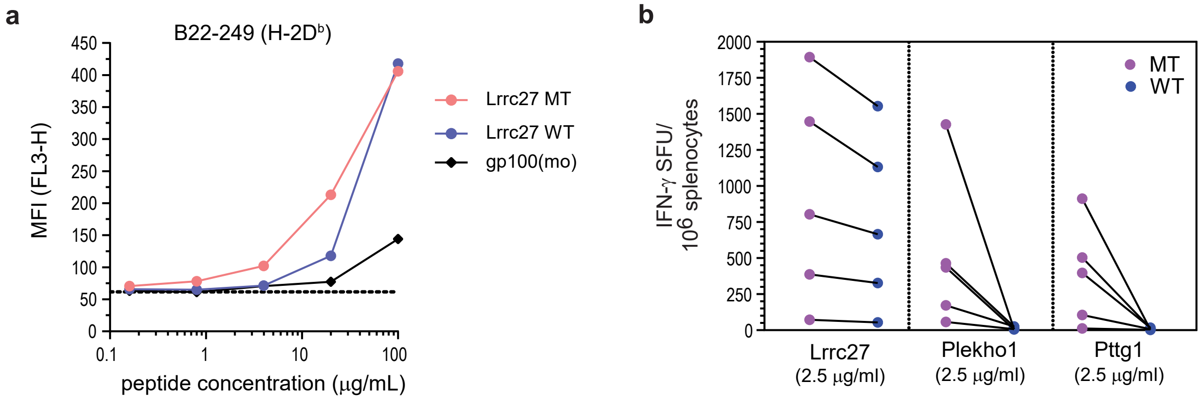

Fig S4. Both MT and WT Lrrc27 peptides bind well to H-2D^b. a *In vitro* peptide-MHC binding assay indicated MT and WT Lrrc27 peptides have equally binding affinity to H-2D^b. Control peptide mouse gp100 (EGSRNQDWL) is known to bind weakly to D^b. The assay was repeated twice and similar results were obtained. **b** Specificity (or cross-reactivity) of immune responses induced by SLP vaccines was assessed by IFNγ ELISpot assay following stimulation with MT or WT peptides. Results from one of the two experiments were shown.
